# Reframing moyamoya vasculopathy within steno-occlusive disease of the circle of Willis

**DOI:** 10.3389/fstro.2026.1890618

**Published:** 2026-09-21

**Authors:** Takahiro Ota

**Affiliations:** Department of Neurosurgery, Tokyo Metropolitan Tama Medical Center, Tokyo, Japan

**Keywords:** genetic susceptibility, moyamoya vasculopathy, phenotypic heterogeneity, RNF213, steno-occlusive disease of the circle of Willis

## Abstract

Moyamoya disease (MMD) is classically defined by progressive steno-occlusion of the terminal internal carotid artery (ICA) with associated basal collateral networks. Historically, however, this condition was conceptualized in Japan as part of a broader anatomical category—steno-occlusive disease of the circle of Willis (SOCW)—which preceded disease-specific nomenclature. Increasing genetic, developmental, and clinical evidence now challenges the strict separation between MMD and moyamoya syndrome (MMS). In this narrative review, we re-examine moyamoya-related conditions within a unified, location-defined framework and propose three advances: (1) an operational definition of SOCW with explicit anatomical boundaries; (2) a spectrum-based classification distinguishing phenotype from etiologic context; and (3) a refined, location-based multi-hit model integrating developmental vulnerability, genetic susceptibility, and acquired triggers. Historical analysis indicates that SOCW was originally defined as an anatomical category, within which moyamoya vasculopathy (MMV) represents the predominant but non-exclusive angiographic phenotype. Genetic studies identify RNF213 as a major susceptibility modifier influencing phenotypic expression and population prevalence, although substantial ethnic heterogeneity and incomplete penetrance indicate that RNF213 is neither necessary nor sufficient for disease development. Additional genetic and environmental factors may further influence disease expression. Developmental factors may contribute to the characteristic localization of disease, although current evidence remains indirect. This SOCW-based framework provides a structured approach to intracranial steno-occlusive disorders, facilitates interpretation of early or atypical presentations, and supports multimodal and longitudinal assessment rather than rigid categorical labeling. By integrating anatomical, genetic, and clinical dimensions, this model offers a pragmatic and biologically grounded basis for future research and refinement of disease classification.

## 1 Introduction

Moyamoya vasculopathy (MMV) is characterized by progressive steno-occlusive alterations affecting the terminal internal carotid artery (ICA) in the region of the circle of Willis, typically accompanied by the formation of abnormal basal collateral networks referred to as moyamoya vessels (Fujimura et al., 2022; Scott and Smith, 2009). Traditionally, this angiographic pattern has been classified as moyamoya disease (MMD) or moyamoya syndrome (MMS) depending on the presence of associated conditions (Scott and Smith, 2009). Although this distinction has been clinically useful, accumulating evidence indicates that it does not fully capture the biological and phenotypic heterogeneity of these disorders.

Radiologically and clinically, MMD and MMS are frequently indistinguishable, particularly at the level of the terminal ICA where disease typically begins (Church et al., 2020; Fujimura et al., 2022; Lee et al., 2014; Scott and Smith, 2009; Zhang et al., 2016). Early-stage cases may present with isolated stenosis without moyamoya collaterals, and unilateral or secondary presentations can progress to bilateral disease over time (Choi et al., 2007; Kim Y. J. et al., 2016). These observations highlight the limitations of phenotype- and etiology-based classification, particularly in early or evolving disease.

Genetic studies further challenge this dichotomy. RNF213 has been identified as a major susceptibility factor, especially in East Asian populations; however, variants are neither universally present nor specific to classic moyamoya phenotypes (Bang et al., 2020; Kamada et al., 2011; Liu et al., 2011; Okazaki et al., 2019). RNF213 is therefore best interpreted as a susceptibility modifier rather than a disease-defining determinant, blurring the boundary between idiopathic and secondary forms.

A defining but unexplained feature of MMV is its consistent localization to the terminal ICA and circle of Willis. Developmental hypotheses, including vascular cephalic neurocristopathy, have been proposed to explain this regional vulnerability, although direct evidence remains limited (Komiyama, 2017; Ota, 2025; Ota and Komiyama, 2021). Nonetheless, the reproducible anatomical distribution of lesions across diverse clinical contexts suggests that MMV is more appropriately conceptualized as a location-defined vasculopathy.

Historically, intracranial steno-occlusive disease in this region was described as steno-occlusive disease of the circle of Willis (SOCW), an anatomical construct that preceded disease-specific terminology (Fujimura et al., 2022; Ihara et al., 2022). Over time, this broader concept became largely subsumed under the moyamoya phenotype, particularly in East Asian clinical practice.

In this review, we reconceptualize SOCW as a location-defined spectrum of intracranial arteriopathy and propose three advances: an operational definition of SOCW with explicit anatomical boundaries, a spectrum-based classification separating phenotype from etiologic context, and a refined multi-hit model integrating developmental vulnerability, genetic susceptibility, and acquired triggers. This framework aims to provide a structured and clinically interpretable basis for understanding moyamoya-related disorders and related steno-occlusive conditions.

This narrative review was based on a structured literature search of PubMed and Scopus (1968–2025) using terms including “moyamoya,” “steno-occlusive disease of the circle of Willis,” and “RNF213,” with selection guided by conceptual relevance.

## 2 Historical background: from steno-occlusive disease of the circle of Willis to moyamoya disease in Japan

Early descriptions of moyamoya in Japan were based on a topographic understanding of intracranial arterial pathology rather than a predefined disease entity. Initial investigations focused on progressive steno-occlusive changes involving the terminal internal carotid artery (ICA) and the circle of Willis, without assuming a single etiopathogenic mechanism. Within this framework, steno-occlusive disease of the circle of Willis (SOCW) was defined as a descriptive anatomical category encompassing heterogeneous non-atherosclerotic intracranial arteriopathies (Kudo, 1968; Nishimoto and Takeuchi, 1968).

This anatomical orientation was reflected in early national consensus documents, which were titled “Guidelines for the Diagnosis of Occlusive Disease of the Circle of Willis” (Ministry of Health and Welfare of Japan, 1977). The subsequent introduction of the term “moyamoya” by Suzuki and Takaku represented a shift toward angiographic characterization, describing the appearance of abnormal basal collateral networks associated with terminal ICA occlusion (Suzuki and Takaku, 1969). Over time, this descriptive term evolved into a disease entity (MMD), driven by the reproducibility and clinical prominence of the moyamoya phenotype, particularly in East Asian populations (Fujimura and Tominaga, 2015; Kuroda and Houkin, 2008).

As a result, the broader SOCW concept was progressively subsumed under the moyamoya framework, leading to an implicit equivalence between SOCW and MMD in clinical practice. In contrast, Western classification systems have generally interpreted moyamoya-like collateral networks as nonspecific responses to chronic arterial occlusion, reflecting differing epidemiological contexts (Ihara et al., 2022; Scott and Smith, 2009). These perspectives have produced a phenotype-centered framework in Japan and an etiology-centered approach in Western practice.

Revisiting SOCW therefore does not introduce a new concept but restores an anatomically grounded framework that can integrate these divergent perspectives. For operational purposes, SOCW in this review is defined as progressive steno-occlusive disease involving the supraclinoid/terminal ICA, proximal ACA (A1), proximal MCA (M1), and posterior communicating artery origin, while excluding isolated distal branch disease and primary basilar atherosclerosis. The proposed operational framework and representative phenotypic categories within SOCW are summarized in Table 1, including classical MMV, early-stage disease without moyamoya vessels, RNF213-associated arteriopathy, overlapping atherosclerotic phenotypes, and secondary forms.

**Table 1:** Proposed operational framework for phenotypic classification and assessment of steno-occlusive disease of the circle of Willis (SOCW).

Proposed SOCW phenotype	Anatomical/angiographic features	Etiologic/genetic context	Suggested assessment
Classical moyamoya vasculopathy (MMV)	Progressive stenosis or occlusion centered on the terminal ICA and CoW, accompanied by characteristic basal moyamoya collateral networks	May occur with or without an identifiable associated condition. Genetic susceptibility, including *RNF213* variants, may modify phenotypic expression	MRA/CTA; DSA when indicated; hemodynamic assessment; evaluation for associated conditions; genetic testing when clinically appropriate
Early or precollateral SOCW	Stenosis or occlusion centered on the terminal ICA/CoW territory without established moyamoya collateral networks	Etiology may be uncertain at presentation; may represent an evolving phenotype preceding collateral formation	Serial vascular imaging; hemodynamic assessment; evaluation for associated conditions and alternative arteriopathies
Non-moyamoya or mixed-pattern SOCW	CoW-centered steno-occlusive disease that does not fulfill the classical MMV phenotype, including lesions with atypical collateralization or overlapping vascular features	Heterogeneous genetic, vascular, inflammatory, or acquired factors may contribute; attribution to a single mechanism may be difficult	MRA/CTA; vessel-wall imaging when appropriate; vascular risk assessment; etiologic work-up; longitudinal imaging

The proposed categories describe vascular phenotypes rather than etiologic disease entities and may evolve or overlap longitudinally. Associated conditions traditionally used to distinguish MMD from MMS are considered etiologic context rather than separate SOCW phenotypes. Likewise, RNF213 status is not considered a distinct SOCW category but a genetic susceptibility modifier that may influence penetrance, age at onset, severity, progression, and phenotypic expression across the SOCW spectrum.

ICA, internal carotid artery; CoW, circle of Willis; MMD, moyamoya disease; MMS, moyamoya syndrome.

## 3 Moyamoya vasculopathy as the dominant phenotype within steno-occlusive disease of the circle of Willis

Within the SOCW framework, moyamoya vasculopathy (MMV) is best understood as the predominant angiographic phenotype rather than a distinct disease entity (Ota, 2025). This distinction clarifies the historical evolution of Japanese diagnostic concepts and addresses ambiguities inherent in the conventional MMD–MMS classification.

### 3.1 MMV as a phenotypic expression rather than a disease definition

MMV is characterized by progressive steno-occlusion of the terminal ICA and circle of Willis with the development of basal collateral networks (Suzuki and Takaku, 1969). Although these collaterals have been regarded as disease-defining features, they more accurately represent adaptive vascular responses to chronic arterial narrowing. The predominance of MMV reflects its epidemiologic frequency and diagnostic visibility, particularly in East Asian populations, rather than a unique underlying pathology (Fujimura and Tominaga, 2015; Kuroda and Houkin, 2008; Liang et al., 2018).

### 3.2 Limitations of the MMD–MMS dichotomy

The distinction between MMD and MMS was introduced to separate idiopathic from secondary cases; however, it does not correspond to consistent differences in vascular pathology (Scott and Smith, 2009; Wang X. P. et al., 2024). Radiologic patterns and disease progression are often indistinguishable, and unilateral or secondary cases may evolve into bilateral phenotypes identical to idiopathic MMD (Church et al., 2020; Fujimura et al., 2022; Lee et al., 2014; Zhang et al., 2016). The dichotomy therefore reflects etiologic labeling rather than biologically distinct vascular processes.

### 3.3 Temporal dissociation between steno-occlusion and moyamoya vessels

A key implication of this framework is the temporal dissociation between arterial narrowing and collateral formation. Early SOCW may present as focal stenosis of the terminal ICA without moyamoya vessels, which develop later as hemodynamic compensation (Choi et al., 2007; Kim Y. J. et al., 2016). These cases are difficult to classify under phenotype-based criteria but can be interpreted within SOCW as early-stage disease, supporting longitudinal assessment over rigid categorization.

### 3.4 MMV as the core phenotype of SOCW

Taken together, MMV represents the dominant but non-exclusive phenotype within SOCW. This spectrum-based perspective accommodates alternative presentations, including early-stage disease and non-moyamoya steno-occlusive conditions. Repositioning MMV within SOCW provides a framework for integrating anatomical vulnerability, genetic susceptibility, and phenotypic variability.

## 4 Genetic contributors: RNF213 as a major susceptibility modifier within SOCW

Genetic susceptibility contributes to SOCW pathogenesis; however, current evidence indicates that genetic factors primarily modulate phenotypic expression rather than define disease identity. Among these, RNF213 is the most consistently associated gene, particularly in populations in which MMV is prevalent.

### 4.1 Population-specific associations

Genome-wide association studies identified RNF213, particularly the p.R4810K variant, as a major susceptibility factor for MMD (Bang et al., 2020; Kamada et al., 2011; Liu et al., 2011). The association is most pronounced in Japanese and Korean patients and is less frequent in Chinese patients, indicating substantial genetic heterogeneity even within East Asia (Cecchi et al., 2014; Liu et al., 2012). The marked geographic distribution of p.R4810K supports a founder effect rather than a universally shared moyamoya-associated variant.

Importantly, p.R4810K is extremely rare or absent in European-ancestry patients with moyamoya angiopathy. Nevertheless, sequencing studies have identified multiple rare RNF213 variants in non-East Asian patients. In European cohorts, these variants are heterogeneous rather than dominated by a single founder allele and preferentially cluster within the C-terminal region encompassing the RING-finger domain. Reported variants include p.D4013N, p.R4062Q, and p.A4399T, although the pathogenic significance of individual rare variants remains incompletely established (Cecchi et al., 2014; Uchino et al., 2025).

Thus, the genetic architecture of moyamoya-related arteriopathy differs substantially across populations: East Asian disease is strongly enriched for p.R4810K, whereas European-ancestry disease is characterized by greater allelic heterogeneity and lacks a comparable major founder variant. This population-dependent variant spectrum indicates that no single *RNF213* variant accounts for the shared vascular phenotype across populations.

### 4.2 Incomplete penetrance and gene-dosage effects

Beyond population-specific variant heterogeneity, RNF213-associated susceptibility demonstrates marked incomplete penetrance (Bang et al., 2020). Many heterozygous carriers remain asymptomatic throughout life, indicating that RNF213 alone is insufficient to determine disease expression. Conversely, increasing genetic burden appears to influence both disease severity and phenotype.

Several studies suggest a gene-dosage effect, whereby homozygous individuals tend to present with earlier disease onset, more severe steno-occlusive changes, and broader vascular involvement compared with heterozygous carriers (Ishigami et al., 2022; Kim E. H. et al., 2016; Mineharu et al., 2022; Miyatake et al., 2012). Homozygous carriers have also been associated with more rapid progression, posterior circulation involvement, and increased frequency of extracranial vascular manifestations, including pulmonary artery stenosis and systemic vasculopathy (Ishigami et al., 2022; Kim E. H. et al., 2016; Ozaki et al., 2022; Miyatake et al., 2012).

Recent evidence further suggests that additional genetic factors may modify disease expression among RNF213 variant carriers. In individuals carrying p.R4810K, the HAPLN3 p.T34A variant has been proposed as a potential modifier associated with increased MMD penetrance, supported by whole-exome sequencing and endothelial-cell experiments. These findings remain preliminary but suggest that the incomplete penetrance of RNF213 may partly reflect interactions with additional genetic modifiers (Xu et al., 2024).

These observations indicate that RNF213 influences disease susceptibility in a quantitative rather than deterministic manner. The relationship between variant burden and phenotype further supports a multifactorial model in which RNF213 modifies the severity and expression of SOCW rather than functioning as a sole disease-defining determinant.

### 4.3 Additional genetic modifiers beyond RNF213

Although RNF213 represents the strongest known susceptibility factor for moyamoya-related disorders, other genetic contributors have also been implicated in moyamoya and related intracranial arteriopathies. Variants affecting pathways involved in vascular remodeling, smooth muscle biology, endothelial signaling, and angiogenesis have been implicated in selected patients with moyamoya and related intracranial arteriopathies (Hitomi et al., 2013; Kim et al., 2020; Miyawaki et al., 2012; Moteki et al., 2015; Uchino et al., 2025).

However, the contribution of these factors appears substantially smaller and less consistently reproduced than that of RNF213. Furthermore, whether such variants function as true susceptibility modifiers within SOCW or reflect distinct disease mechanisms remains uncertain. Rather than acting as independent determinants, these factors may influence vascular remodeling capacity and contribute to interindividual variation in disease severity and phenotypic expression.

Within the SOCW framework, genetic susceptibility is therefore likely to involve multiple interacting modifiers rather than a single disease-defining gene.

### 4.4 Broader phenotypic associations

RNF213 variants are also associated with non-moyamoya intracranial arteriopathies, including intracranial atherosclerotic disease and large-artery ischemic stroke (Bang et al., 2020; Kim et al., 2020; Miyawaki et al., 2012, 2013; Liu and Gutierrez, 2020).

### 4.5 Functional insights

RNF213 encodes a protein with ATPase and ubiquitin ligase activity implicated in angiogenesis, endothelial function, and vascular remodeling (Hitomi et al., 2013; Ohkubo et al., 2015). Emerging data also indicate roles in immune and inflammatory pathways, including interferon signaling and host defense (Otten et al., 2021; Ozaki et al., 2022; Tan et al., 2025; Thery et al., 2021). These functions support a model in which RNF213 acts as a context-dependent modifier that amplifies vascular vulnerability under inflammatory or environmental stress.

### 4.6 Conceptual positioning of RNF213 within SOCW

Collectively, these findings position RNF213 as a major, but non-exclusive, susceptibility modifier within SOCW. Its population-specific prevalence explains the dominance of MMV in East Asia, whereas its incomplete penetrance and broader phenotypic associations preclude its use as a defining criterion. Instead, RNF213 should be interpreted within a multifactorial framework in which genetic susceptibility interacts with anatomical and environmental factors to shape disease expression.

However, genetic factors alone do not account for the consistent anatomical localization of SOCW, necessitating consideration of additional mechanisms underlying regional vascular vulnerability.

## 5 Developmental topography: why the terminal ICA region and circle of Willis?

A consistent feature of SOCW is its non-random anatomical predilection for the terminal ICA and circle of Willis. This localization is not readily explained by systemic risk factors alone. While genetic susceptibility, including RNF213, modulates disease expression, it does not account for this regional specificity, suggesting that additional mechanisms may contribute to location-dependent vulnerability.

### 5.1 Developmental heterogeneity of vascular wall components

Developmental studies indicate that mural cells, including vascular smooth muscle cells and pericytes, arise from heterogeneous embryological origins. In the craniofacial and forebrain vasculature, cephalic neural crest cells contribute to mural cell populations in a regionally restricted manner, implying the existence of developmental boundaries in vascular wall composition (Etchevers et al., 2001; Sinha et al., 2014). Such heterogeneity may influence vascular phenotype and response to injury. However, direct lineage mapping of human cerebral arteries remains limited, and the relevance of these findings to SOCW should be interpreted cautiously.

### 5.2 Developmental assembly and hemodynamic vulnerability

The circle of Willis develops through complex vascular remodeling processes involving dynamic changes in flow patterns and vessel regression or persistence (Menshawi et al., 2015). This process results in substantial anatomical variability and may predispose the terminal ICA region to localized hemodynamic stress, particularly at bifurcation points. Such conditions may lower the threshold for steno-occlusive remodeling in susceptible individuals. This perspective is consistent with the observation that early-stage SOCW may present as focal stenosis without moyamoya vessels, with collateral formation occurring as a secondary adaptive response (Choi et al., 2007; Kim E. H. et al., 2016).

### 5.3 Developmental topography as a hypothesis

The concept of vascular cephalic neurocristopathy has been proposed as a possible explanation for the preferential involvement of the terminal ICA region (Komiyama, 2017). While this hypothesis provides a coherent developmental framework, it remains unproven and should be regarded as one of several potential explanations. The observed correspondence between lesion distribution and neural crest–related vascular territories may reflect causal vulnerability or coincidental overlap. At present, no direct evidence demonstrates that neural crest–derived vascular components selectively predispose these arteries to disease in humans.

### 5.4 Integration with genetic susceptibility

Taken together, developmental factors may contribute to defining the anatomical territory in which SOCW occurs, whereas genetic factors such as RNF213 influence susceptibility and phenotypic expression. This interpretation supports a model in which location, genetic predisposition, and environmental influences interact to shape disease manifestation, without assigning primary causality to any single factor.

## 6 Reframing moyamoya disease and moyamoya syndrome within steno-occlusive disease of the circle of Willis

The preceding analyses indicate that the conventional distinction between moyamoya disease (MMD) and moyamoya syndrome (MMS) is conceptually limited. Within the SOCW framework, both entities are more appropriately interpreted as context-dependent manifestations of a shared, location-defined arteriopathy.

The MMD–MMS dichotomy was originally introduced to distinguish idiopathic cases from those associated with identifiable conditions (Scott and Smith, 2009). However, this distinction does not correspond to consistent differences in vascular pathology. Angiographic features and clinical progression frequently overlap, and secondary or unilateral cases may evolve into phenotypes indistinguishable from idiopathic MMD (Kim Y. J. et al., 2016; Kuroda and Houkin, 2008; Suzuki and Takaku, 1969). These observations suggest that the distinction primarily reflects etiologic context rather than intrinsic vascular biology.

From a pathoanatomical perspective, SOCW provides a common structural framework within which these phenotypes arise. Genetic susceptibility, including RNF213, and associated conditions influence disease expression, but do not alter its characteristic localization to the terminal ICA region. This interpretation accommodates both RNF213-negative cases and secondary arteriopathies while preserving anatomical consistency.

Accordingly, moyamoya vasculopathy should be regarded as a shared angiographic phenotype within SOCW rather than as a disease-specific marker. This reframing supports a shift toward anatomically grounded classification, in which etiologic variation is interpreted as a modifier of disease expression rather than as the basis for categorical separation.

## 7 Diagnostic and clinical implications of the SOCW-based framework

Reframing moyamoya-related disorders within the SOCW framework primarily refines diagnostic interpretation and longitudinal assessment rather than altering established treatment strategies. By emphasizing anatomical localization and temporal evolution, this approach provides a structured basis for interpreting early and atypical presentations. The proposed framework for phenotypic classification, etiologic and genetic contextualization, and longitudinal assessment of SOCW is summarized in Figure 1.

**Figure 1:**
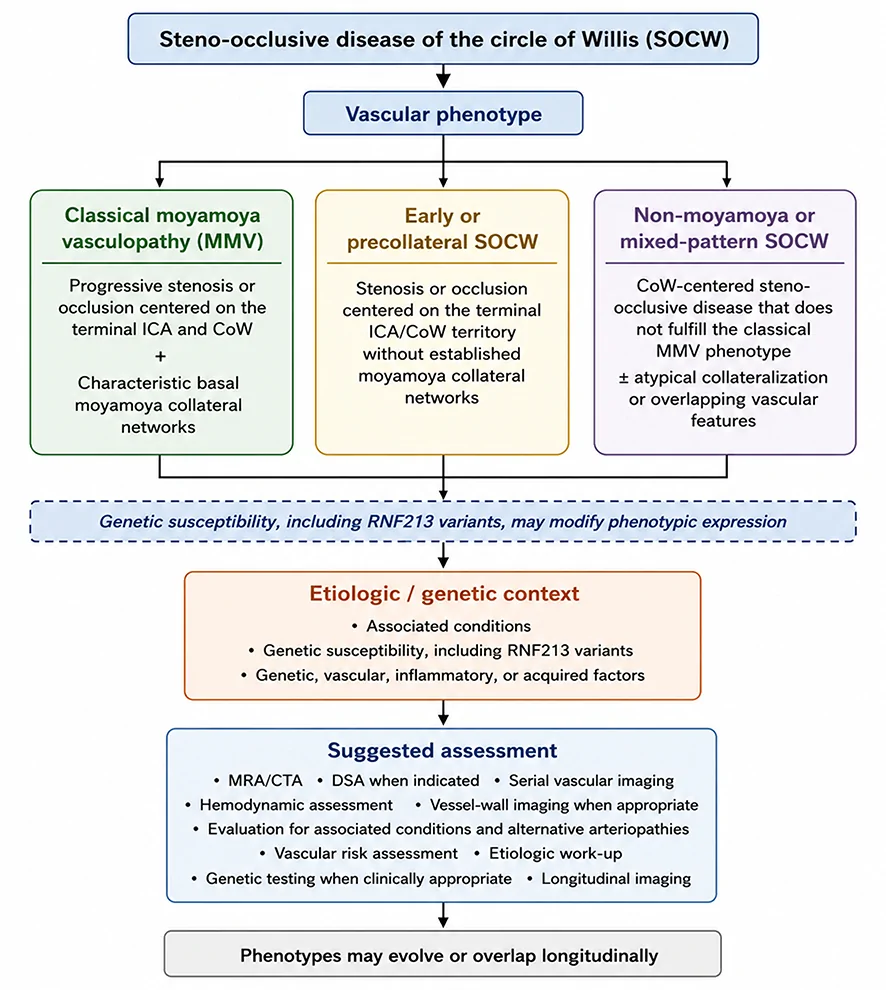
Conceptual framework for evaluating steno-occlusive disease of the circle of Willis (SOCW). SOCW is first characterized by vascular phenotype into three categories. Phenotypic expression is influenced by etiologic and genetic context, including genetic susceptibility factors such as *RNF213* variants, and requires comprehensive assessment. Longitudinal reassessment is important because phenotypes may evolve or overlap over time.

### 7.1 Early-stage disease recognition

Early-stage disease often presents as focal stenosis or occlusion of the circle of Willis without visible moyamoya vessels (Choi et al., 2007; Kim Y. J. et al., 2016; Wang J. et al., 2024). Such cases are often difficult to classify under phenotype-based criteria. Within the SOCW framework, they can be interpreted as early-stage disease within a location-defined arteriopathy, with collateral formation representing a secondary and temporally variable response. This perspective supports early recognition and reduces diagnostic uncertainty at initial presentation.

### 7.2 Integration of etiologic and genetic information

Although associated conditions and genetic findings remain clinically relevant, they should be interpreted in the context of shared vascular pathology. Radiological patterns and disease evolution are often similar between idiopathic and secondary cases (Guey et al., 2015; Scott and Smith, 2009; Velo et al., 2022; Wang X. P. et al., 2024). RNF213 testing may provide prognostic insight, particularly in East Asian populations; however, it should not be used as a diagnostic determinant. Rather, RNF213 status functions as a modifier of susceptibility and disease course, and should be integrated with anatomical and imaging findings.

### 7.3 Diagnostic approaches and emerging biomarkers

The SOCW framework does not introduce disease-specific diagnostic tests but instead emphasizes integration of existing anatomical, hemodynamic, and genetic assessments. Conventional vascular imaging, including MRA, computed tomography angiography, and DSA, remains central for identifying steno-occlusive lesions and collateral formation. High-resolution vessel wall MRI may provide additional information regarding arterial remodeling and vessel wall characteristics (Waddle et al., 2022; Yamamoto et al., 2019).

Hemodynamic evaluation using techniques such as arterial spin labeling, perfusion MRI, positron emission tomography, or single-photon emission computed tomography remains important for assessing functional disease severity and guiding treatment decisions.

At present, no biomarker is sufficiently specific for SOCW or MMV diagnosis. Genetic testing for RNF213 variants may provide susceptibility and prognostic information, particularly in East Asian populations, but does not constitute a diagnostic marker. Emerging candidates—including circulating inflammatory mediators, endothelial markers, microRNAs, and angiogenic factors—have shown preliminary associations with moyamoya-related disorders; however, their clinical utility remains unestablished (Ohkubo et al., 2015; Otten et al., 2021; Tan et al., 2025; Thery et al., 2021).

Within the SOCW framework, future diagnostic strategies will likely depend on multimodal integration rather than a single disease-defining biomarker.

### 7.4 Implications for clinical decision-making

The SOCW framework does not, at present, modify established indications for revascularization. Treatment decisions should continue to be based on clinical presentation, disease progression, and objective evidence of hemodynamic compromise rather than on the SOCW label itself. Surgical revascularization remains the principal treatment for symptomatic MMV with impaired cerebral perfusion or cerebrovascular reserve, whereas the role of prophylactic intervention in asymptomatic or minimally symptomatic disease remains less clearly established.

The potential clinical value of the SOCW framework may instead lie in the precollateral stage. Stenosis centered on the terminal ICA or circle of Willis without established moyamoya vessels should not automatically be regarded as unrelated or nonspecific simply because conventional angiographic criteria for MMV are not yet fulfilled. When such lesions occur in a characteristic distribution, particularly in the absence of conventional atherosclerotic features, longitudinal vascular imaging and hemodynamic assessment may help identify progression toward a classical MMV phenotype. Genetic susceptibility, including RNF213 status, may provide additional context but should not independently determine treatment.

Thus, recognition of precollateral SOCW may justify closer surveillance but does not itself constitute an indication for surgical intervention. Evidence of progressive stenosis, emerging collateralization, recurrent ischemic symptoms, or deterioration of cerebral perfusion or cerebrovascular reserve would provide stronger grounds for reconsidering management. This distinction allows the SOCW framework to contribute to earlier recognition and longitudinal risk assessment without extending current treatment recommendations beyond the available evidence.

### 7.5 Longitudinal assessment

SOCW-related disorders are dynamic, with progression, stabilization, or phenotypic transition occurring over time. Serial imaging and functional assessment are therefore essential. Emphasizing anatomical territory and temporal evolution provides a consistent framework for follow-up and may facilitate future refinement of risk stratification as genetic and imaging data accumulate.

## 8 Limitations and future perspectives

Although the SOCW-based framework provides a coherent anatomical context for moyamoya-related disorders, several limitations warrant consideration.

### 8.1 Evidence and methodological constraints

First, evidence supporting developmental vulnerability and vascular cephalic neurocristopathy remains largely inferential. While lineage-tracing studies suggest region-specific contributions of neural crest-derived cells, direct validation in human cerebral arteries is limited, and spatial concordance cannot be interpreted as causative (Etchevers et al., 2001; Sinha et al., 2014). Alternative explanations, including regional hemodynamic characteristics, differences in vascular wall structure, and local biological responses, may also contribute to the characteristic localization of lesions.

Second, genetic association studies—including those involving RNF213—are influenced by population structure, incomplete penetrance, and phenotypic heterogeneity (Cecchi et al., 2014; Mineharu et al., 2022). The strong association observed in East Asian populations may not be generalizable to other populations, and the relationship between genotype and vascular phenotype remains incompletely understood.

Third, SOCW itself should be regarded as a conceptual and operational framework rather than a definitive biological entity. The proposed anatomical boundaries remain partially arbitrary, and overlap with other intracranial arteriopathies—including intracranial atherosclerotic disease and isolated branch-vessel disorders—may occur in selected cases. Accordingly, future validation using prospective clinical, genetic, and imaging studies will be necessary to determine whether SOCW represents biologically distinct subgroups or a broader continuum of vascular remodeling.

Current classification systems also rely heavily on angiographic morphology and cross-sectional assessment, potentially overlooking the dynamic and temporally evolving nature of disease within the circle of Willis (Kamada et al., 2011; Okazaki et al., 2019). Longitudinal imaging, high-resolution vessel wall studies, and hemodynamic modeling are needed to clarify mechanisms of primary remodeling and collateral adaptation (Waddle et al., 2022; Yamamoto et al., 2019).

### 8.2 A location-based multi-factorial model

The low penetrance of RNF213 variants supports a multifactorial model of disease pathogenesis. Within the SOCW framework, this can be conceptualized as an interaction between location-defined vulnerability, genetic susceptibility, and additional modifying factors (Figure 2). Developmental and anatomical features may define the disease-prone territory, genetic factors such as RNF213 influence susceptibility, and environmental or inflammatory processes act as triggers of disease onset and progression.

**Figure 2:**
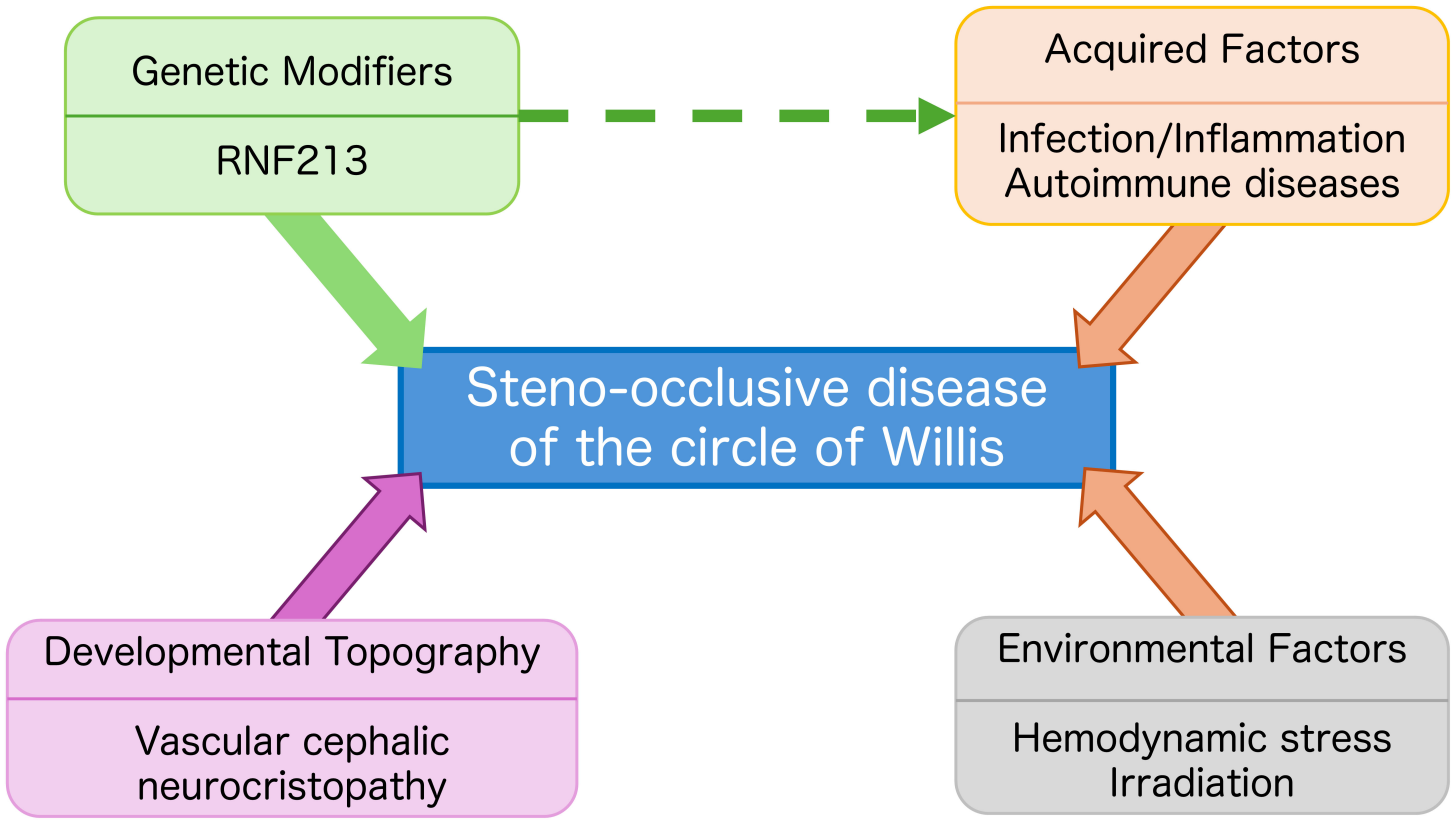
*Phenotypic expression within SOCW is shaped by multiple interacting determinants*. Developmental topography, potentially linked to region-specific mural cell lineages derived from cephalic neural crest cells, may confer a pre-existing anatomical susceptibility of the terminal ICA region. Genetic susceptibility, including RNF213 variants, acts as a context-dependent modifier, influencing penetrance and phenotypic predominance, whereas acquired factors, such as immune-mediated or inflammatory processes, irradiation, and hemodynamic stress, may serve as subsequent triggers that modulate disease onset and progression. The relative contribution of each factor may vary across individuals and populations.

This formulation separates lesion localization from genetic determinants and provides a coherent explanation for anatomical specificity, phenotypic variability, and incomplete penetrance. However, it remains hypothetical and requires validation through integrated genetic, developmental, and longitudinal studies.

### 8.3 Future directions and conceptual refinement

Future research should emphasize location-centered and longitudinal approaches that extend beyond classic moyamoya phenotypes. Inclusion of RNF213-negative cases and non-moyamoya steno-occlusive disease may help identify shared mechanisms that are not apparent in phenotype-based classifications. Prospective integration of vascular imaging, hemodynamic assessment, genetics, and molecular profiling will be important for determining whether the proposed SOCW phenotypes represent biologically meaningful subgroups and for identifying markers of disease progression.

At present, treatment of MMV remains directed primarily toward prevention of ischemic or hemorrhagic events rather than correction of the underlying molecular abnormality. Surgical revascularization remains the principal treatment for symptomatic or hemodynamically significant disease, and no disease-modifying therapy specific to SOCW has been established. Similarly, no clinically available gene therapy currently targets RNF213 variants. Experimental approaches using patient-derived induced pluripotent stem cells and gene-editing technologies have provided platforms for investigating the functional consequences of RNF213 variants and may facilitate future identification of therapeutic targets; however, these approaches remain preclinical.

The SOCW framework should therefore be regarded as an iterative model rather than a fixed taxonomy. Its value lies in providing an anatomical basis for integrating emerging genetic, biological, imaging, and therapeutic insights as evidence accumulates. Future therapeutic development may ultimately focus not on a single genetic variant but on downstream mechanisms shared across SOCW phenotypes, including abnormal vascular remodeling, endothelial dysfunction, and inflammatory signaling.

## 9 Conclusions

Steno-occlusive disease of the circle of Willis (SOCW) provides a location-defined framework that unifies disorders traditionally classified as moyamoya disease (MMD) and moyamoya syndrome (MMS). Within this framework, moyamoya vasculopathy (MMV) is best understood as a predominant, but non-exclusive, angiographic phenotype rather than a discrete disease entity.

Genetic and developmental evidence supports this reconceptualization. RNF213 acts as a susceptibility modifier rather than a defining determinant, and may influence phenotypic expression without explaining the consistent anatomical localization of disease.

Accordingly, the MMD–MMS distinction reflects etiologic context rather than fundamental vascular biology. A location-based, multi-factorial model—integrating anatomical vulnerability, genetic susceptibility, and additional modifiers—offers a coherent explanation for phenotypic variability and disease evolution.

This framework provides a structured and clinically interpretable basis for classification and future investigation of intracranial steno-occlusive disorders.
